# Considerations on Alterations of the Blood in Different Diseases

**Published:** 1834-04-01

**Authors:** 


### Considerations on some of the Alterations which the Blood undergoes in different Diseases. By *M. Roche.*

[Journ. Hebdom.]

This curious subject of pathology has of late, and especially for the last quarter of a century, been much overlooked; but although savouring, as it does, of the long-exploded doctrines of the ancient humorism, we think that we can perceive signs of an approaching more zealous investigation of its phenomena. M. Lecanu has shewn that the blood of icteric patients contains the yellow and blue colouring principles of the bile, and that there is sometimes a simultaneous deficiency in the quantity of the proper red colouring matter. MM. Prevost and Dumas have discovered urea in the blood of animals, from which the kidneys were extirpated, and we may rationally infer that the same chemical changes may ensue, when these organs become much disorganized in texture from disease. Many poisonous substances, also, introduced into the circulation, may be quickly detected in the urine. M. Mondezert, in some late researches on the serum of the blood, has arrived at the conclusion, that the inflammatory crust of the blood in certain diseases is derived from the serum, and not from the crassamentum, and that, therefore, it is probably formed by concreted albumen.

The physical characters of blood which may have undergone some important changes, are often little calculated to throw light on the specific nature of these changes. Sometimes, indeed, it is more fluid than natural, and is, as it were, dissolved; at other times it is more consistent, or is grumous, or has a pitchy character, or is black, greenish, decomposed, or, again, it may have too much serum, or too much fibrine; but, as yet, these changes, however important, have not hitherto been sufficiently examined in their relations to particular diseased conditions, to enable us to apply our knowledge to any purposes of therapeutics. As a general remark, the blood is fibrinous in the phlegmasiæ—serous in chlorosis and in anæmia—black and grumous in scurvy—pitchy and uncoagulable in the pestilential cholera, and deficient in animal matter in genuine diabetes.

The causes of the changes of the blood are generally referable to the absorption of miasmatous or poisonous agents, which may arise either from the decomposition of animal and vegetable substances in a state of putrefaction, or from individuals affected with contagious diseases; 2dly, of purulent or putrid matters, or of specific viruses, whether of an animal, vegetable, or mineral nature; and, thirdly and lastly, of crude and ill-prepared chyle, in consequence of unsoundness or insufficiency of the food. Now, in the study of these alterations, the first general observation we make is, that some of them consist simply in the presence of a noxious agent in the circulating mass, while, in others, there is a positive modification induced in its chemical composition. It is, therefore, of importance to distinguish these two modes of morbid or abnormal change in our present investigation. We have been taught by the results of numerous experiments to believe, that the injection into the veins of putrid matters, derived from the decomposition of animal and vegetable matters, gives rise to diseases perfectly analogous to those induced by the absorption of certain miasmata; also, that the direct injection of pus into the blood is followed by symptoms very similar to those which arise from its spontaneous absorption from the surface of wounds, abscesses, and so forth, especially when these have become suddenly dried up; that the inoculation of the putrid sanies of a carbuncular or hospital gangrenous ulcer, will cause the development both of the local and of the constitutional symptoms of these kinds of sore; and, lastly, that the introduction of almost all the very active medicinal or poisonous drugs into the circulation, whether they be directly injected, or permitted to be slowly absorbed from the skin, induces nearly the same effects as follow their being admitted into the stomach. But all these facts, however curious, interesting, and instructive, do not make us acquainted with a single direct alteration of the blood; they do not even prove that this fluid undergoes any change in its own composition; and, correctly speaking, the only logical inference which may be drawn from them is, that the blood is the vehicle for the reception and transmission of extraneous matters, howsoever they have become mixed with it. This view of the subject is corroborated by the observation, that the greater number of the active substances thus introduced into the blood become eliminated, "en nature," at some one of the excretory organs; and that, if the dose or quantity be small, and not very potent, their effects cease, and they become gradually thrown off from the system: from this we may conclude, that the poison or virus was only mixed, and not incorporated or assimilated with the blood; and, consequently, that this fluid did not experience any decided chemical change in its composition. Nevertheless, while we admit that, in those diseases which are induced by the absorption or injection of noxious substances into the blood, there are no positive proofs of any consequent alteration (although the contrary is not ascertained, viz. that no alteration does actually take place), it is still quite proper to arrange among the diseases of the blood, all such as exhibit analogous phenomena with those which we can at will induce in our experiments. The observation of pathological facts, and a cautious, yet unfettered, interpretation of the data which they afford, are calculated to throw more light upon the question of the changes of the blood, than has hitherto been afforded by examining the physical and chemical characters, or even by direct physiological experiments. This last method of investigation lends, however, upon all occasions, a valuable assistance to clinical enquiries, and there is not, perhaps, any subject which it has illustrated so satisfactorily as that which we are at present considering.

In all diseases which become developed in the midst of infected foci, such as marsh intermittent fevers, typhus, yellow fever, the plague, and the pestilential cholera, the primary element, or first link in the chain of the disease, is a change or infection of the circulating mass. That marsh fevers are owing to the agency of certain miasms which are generated by the decomposition of wet putrefying animal and vegetable substances, few will be disposed to doubt. Chemistry indeed has hitherto failed in disclosing to us the nature of these miasms; but this we know, that by exposing animals to the effect of water, which has been charged with them, we may produce at will intermittent fevers, [Rigaud de l'Isle]; and often it is only necessary to withdraw them from the infected medium, for the morbid effects speedily to cease. We are therefore quite justified in asserting that miasms are the direct cause of marsh fevers. Typhus also is often traceable to the operation of a similar cause;—thus we see it generated in close chambers, where too many are confined, and, upon many occasions at least, it is obviously propagated by emanations from a sick to a healthy person. It is easy to prove that the principal cause of the yellow fever exists in the very places, where it is known to be most frequent and fatal, and that in truth it arises from a miasm generated in these localities. As to the plague, all authors agree in attributing to it an analogous origin; and its unequivocally communicable character confirms this etiology; and with respect to the cholera, we think that we may very safely and fairly affirm, that it is the result or effect of some unknown principle, developed in, or perhaps only diffused through, the atmosphere. Now there is but one way or mode of introduction into the body of all these morbific agents, and that is by absorption;—they become blended in truth with the blood, and their first effect is to induce some change or other in its properties, either by a simple admixture with its constituents, or by a direct action upon its composition.

The study of the symptomatology of these diseases illustrates and confirms the etiological speculations which we have briefly noticed. They all exhibit in the same order, although in very different degrees, a succession of morbid phenomena or phases, which seem to correspond exactly to the development of the morbific principle within our bodies, from the period of its introduction to that of its elimination and expulsion. A general malaise is usually the earliest symptom, and indicates the absorption of the poison into the system: then follows certain nervous feelings, such as shiverings, sensation of cold all over the surface, head-ache, delirium, cramps, and other convulsive movements; proceeding probably from the poison having been conveyed by the circulating torrent to the principal nervous centres, which, according as the impression made upon them is of an irritating or of a benumbing nature, give rise to symptoms either of excitement, or of stupor, or possibly of a blending of the two. After a lapse of time, which must necessarily vary with the excitability of the patient, and with the dose and activity of the operating poison, there supervene symptoms of general disturbance, such as change of pulse, heat of skin, intense thirst, altered urine, starting of the tendons, restlessness, and anxiety; by the occurrence of which phenomena, we infer that the miasm has been brought in contact with all the organs of the body, and that they have begun to react against its deleterious effects. The symptoms of this stage, viz. that of reaction, vary according to the nature of the morbific cause ; and as different poisons exhibit great diversity in their special, and what we may call their characteristic mode of destroying life, these symptoms to which we allude differ in each case, as well in respect of the order of their succession, as of their severity and complication. When the reaction is violent, either simple synocha, or synocha associated with a local inflammation, is developed. Lastly, the fourth stage or set of symptoms set in; and this is characterised by profuse sweats, or by vomitings, purging, the discharge of turbid and offensive urine, by a jaundiced state of the skin, an eruption of petechiæ, miliary vesicles, or of external glandular phlegmons, and sometimes of local mortifications. Every appearance at this period attests, an effort of the system to eliminate and carry off the morbific principle, by some of the great emunctories, as the skin, gastro-intestinal, or urinary passages. Now if we attend to the symptoms which those animals which are directly subjected in our experiments to the operation of a poison exhibit, we find that they follow nearly the same succession as we have just now described;—a general malaise immediately, or very quickly follows the injection of any noxious substance into the veins; to this malaise succeeds the nervous disturbance; this again is speedily associated with a general constitutional reaction; and then the system tries to get rid of the poison by vomiting, purging, or sweating. It is indeed true, that in numerous cases of poisoning, these stages or successive series of phenomena are obscurely marked, and cannot be very satisfactorily distinguished from each other; or that the disease may be suddenly cut short, or may prove fatal after the formation of the first or the second set of symptoms;—if the reaction be very violent and the intoxication be feeble, the evil aborts from its commencement; and, on the contrary, when the reaction is feeble, and the intoxication is severe, death may very speedily or even instantaneously follow. Experience supplies us with numerous illustrative examples of both these conditions, and of various intermediate shades, verging more or less to one or to the other tableau which we have described; but still, as a general remark, we may repeat that the common or ordinary march of all such diseases as are induced by miasmata and other poisonous agents, is progressive from one stage to another, in the mode explained above. Now an attentive examination of this subject infallibly leads us to anticipate an infected or altered state of the blood; and the circumstance of the communicability of most of these maladies renders our suspicions still more probable; but as it may be objected to this argument that we are assuming what has not been proved, and is not admitted by many physicians, viz. the property of propagating and transmitting themselves from one person to another, it will be proper to consider the force of the objection, before we proceed further in our inquiries. By the term "contagion," we mean that feature or character of certain diseases to multiply themselves by being communicable, either by means of direct contact, or through the medium of the atmosphere conveying the effluvia from one body to another. At first sight, nothing might seem more easy of solution than the question, what diseases are contagious and what are not; and yet few subjects of pathology have been more keenly disputed. Every one, it is true, is agreed as to the contagious properties of the itch, syphilis, small-pox, and hydrophobia; because these diseases may be induced at will by direct inoculation of their specific poisons, and we have daily proof of such occurrences. The proofs, too, of the contagion of measles, of scarlatina, and of hooping-cough, are by most considered to be quite conclusive; but very discordant are the sentiments of authors as to the communicable property of miasmatic diseases, putting, as a matter of course, out of the question pure intermittent fevers, which have very rarely been considered as directly contagious. Many of the best observers furnish us with numerous examples, to prove that typhus, yellow fever, the plague, and also the pestilential cholera, are communicable, at least occasionally; but their conclusions have been gainsayed by an equal host of authorities, and these, too, equally worthy of credit. The opinion to which we have been led by a rigorous comparison of what has been adduced by both parties is, that these last-mentioned diseases are not absolutely, essentially, and invariably contagious, but that, under some circumstances, they appear to be so, while, under others, they exhibit no such character. There has been more discrepancy of sentiment in reference to the contagion of yellow fever and of cholera, than of the plague and of typhus; indeed, with respect to these latter two, most physicians are inclined to admit that they are occasionally, at least, if not always, communicable; and this very admission strongly predisposes us to believe, that the yellow fever and cholera may also be so, from the simple circumstance of the striking analogy which all these four maladies exhibit in many of their features; such as their origin from some atmospheric cause, and the course or march of their symptoms; it is from these very characters of resemblance, that we are induced to suppose that there is a similar analogy in their effects. The results of physiological experiments add strength and probability to this supposition—they teach us that all substances injected into the veins, or absorbed from any surfaces, pass unchanged, "en nature," into the circulating mass—that they impregnate, "en nature," every organ, and that, finally, they become eliminated, "en nature," by some of the leading emunctories. Analogy, facts, and reasoning shew that typhus, yellow fever, the plague, and the cholera, are produced by the absorption of some principle or morbific agent, differing probably in each of these maladies, diffused through the atmosphere. Now this agent, like camphor, phosphorus, alcohol, &c. may be supposed to pass, "en nature," into the blood, and to be expelled "en nature," if the constitution be sufficient for the effort; and some of the phenomena of the cholera render this supposition very probable; all the excretions of a patient labouring under this disease, as the cutaneous and pulmonary transpirations, the matters rejected by vomiting and by stool, have a similar and very characteristic odour, and the two last have exactly the same appearance. A similar remark is applicable to some of the other miasmatic maladies.

If, then, it be actually the exciting agent of the disease which makes its escape, "en nature," through one or through several of the excretory organs, we can easily understand how this, when absorbed into a healthy constitution, may give rise in it to the repetition of the same effect which had already taken place in another constitution. The effect is not, indeed, inevitable and constant, but is partly dependant upon a multitude of accessory circumstances, such as the existing temperature, the close or free state of the atmosphere, the constitutional condition of those exposed to the miasm, and the activity or virulence of the miasm itself. From all these considerations it follows, that theory might lead one to admit the possibility of a contagious property in all miasmatic diseases, including even marsh fevers, and we shall find that numerous facts favour the idea; but, before adverting particularly to these, we should observe, en passant, that the contagionists themselves have grievously injured their own cause, by assuming that, if a disease can be proved to be at any time, and under any circumstances, communicable, it must be absolutely, essentially, and invariably so upon all occasions; and by being, in consequence of this speculative fatalism, too ready to admit the correctness of all facts which seemed favourable to their doctrines. However this may be, if we have succeeded in establishing that all miasmatic diseases may propagate themselves by contagion, we shall have added another argument to what we have above said respecting their nature, to wit; that they all occasion an infection or change in the condition of the circulating mass; and this was the chief object which we had in view. We are well aware that many will immediately impugn such a conclusion, and will tell us that intermittent fevers, typhus, the yellow fever, the plague, and the pestilential cholera have often, and do often, appear in sporadic cases, and under circumstances where little chance of any miasmatic taint could possibly exist, and, as a logical corollary from these data, they state that, if any of these maladies ever arise spontaneously, and without any miasmatic cause, we are bound to suppose that they may upon all occasions do so; and that, therefore, the admission of any such morbific principle is perfectly gratuitous and unwarranted. But let us not be led astray by the apparent force of a syllogism; let us remember that, when a disease does present itself only sporadically, we are not justified in thence inferring that it must have arisen spontaneously, or, more correctly speaking, fortuitously, that is, without the operation of its ordinary exciting cause. Is it not conceivable that miasms, which are generally admitted to be the primary causes of most epidemics on a large scale, may occasionally be generated only sparingly and locally, and thus give rise to insulated cases of the diseases? As a mere question of probabilities, we think that there can be but little dispute, that the doctrine which maintains that all cases, the insulated as well as the epidemic, of what we have designated miasmatic diseases, arise solely and exclusively from the operation of some external morbific principle, is more consistent with facts and with theoretical reasoning than the opposite doctrine, that they are all, and upon all occasions, essentially independent of any such principle or agent.

By far the greater number of cases of poisoning belong to the class of infections or alterations of the blood. It may possibly be objected, that the effects of the caustic poisons, or of the strong acids and alkalis, cannot be referred to such a catalogue. We admit the truth of the objection; but the force of it can scarcely be said to reach our position, for it is quite incorrect to include the effects of these virulent chemical agents, under the term of poisoning or intoxication, seeing that they act only as local caustics or corrosives, and that the constitutional disturbance which quickly supervenes to their being swallowed is owing, not to the absorption of any portion of them into the blood, but merely from the intimate and co-operating sympathy of almost every organ with the stomach and intestines. Very different are the constitutional effects of a poisonous dose of opium, of aconite, or even of arsenic; and yet this last agent agrees so far with the acids and alkalis, in being a virulent corrosive. In many cases of genuine poisoning, we can discover in the blood the particular poison which we administered to the subject of our experiments; and the successive series of symptoms very nearly coincide with the march or course of a miasmatic disease: first of all, there is the stage which indicates the absorption of the poison—then follows that of its being brought in contact with the nervous centres—next is the stage of the sanguineous re-action, and, lastly, that of the elimination and expulsion of the peccant cause.

Another set of diseases which we arrange under the head of alterations of the blood, are small-pox, measles, scarlatina, hooping-cough, hydrophobia, the effects of bites of poisonous animals, and some will add, syphilis, scabies, &c. As to the first six, there can be but little difference of opinion on the correctness of our classification, since in all of them there occurs an evident inoculation of a virus, an absorption of this virus, the consequent development of constitutional symptoms (among which are almost always present, certain disturbances of the nervous functions, arising, as we suppose, from the direct contact of the virus with the nervous centres), and finally an eliminating effort, rendered apparent by cutaneous eruptions, by sweating, salivation, &c. Whenever this quadruple succession of stages is observed, we are inclined to suppose, that the disease depends upon some poisonous agent received into the system, and upon a consequent change of some sort or another, induced in the circulating mass. The particular physiognomy of the case is determined by the nature of the morbific principle, just in the same way as we observe to take place in cases of poisoning, whose symptoms though they may have a general resemblance, vary according to the poison which has been administered. But syphilis and scabies have no other features which ally them to the preceding diseases, except their contagious property. Neither of them exhibit those symptoms which we have pointed out as indicating an infection of the blood; and from this negative character alone, we conclude, that they ought not to be classed along with the others. Scabies appears to be limited to the cutaneous tissue; and syphilis has probably its seat in the lymphatic system; but this is only conjectural.

Important therapeutic results may be derived, we think, from our manner of contemplating the alterations of the blood, when induced by infection, or intoxication of its mass. The first indication in their treatment ought to be to relieve, as far as it is possible, the system of the presence of the morbific matter; this is to be done by bleeding, by vomiting and purging, and by exciting the perspiratory discharge: by these means we shall sometimes succeed in evacuating the greater part of the noxious matter, and the effect of what remains behind is in consequence enfeebled and modified. The second indication is to obviate and neutralize the immediate specific operation of the poison: the third is to combat the effects produced by it upon the principal organs, whether these effects be sthenic, asthenic, or of an intermediate character, by different remedies suited to the nature of each case; and finally we ought to encourage and favour its expulsion from the system. We must never confine our treatment merely to the fulfilment of one of these indications, to the exclusion of the others; and the circumstance of the same treatment being not applicable to all the stages of the case, is one reason among others, that very different, or even very opposite indications have been approved of and practised by equally respectable authorities. There is only one truly preservative means, against those diseases which depend upon an infection of the blood; and that is, the removal from the locality in which they are generated;—all other hygienic precautions are often quite impotent unless this be done. Even when the disease has actually commenced, its virulence may be considerably mitigated if the focus of infection be left. Attention, therefore, to this rule must be always of great importance until the happy discovery is made of special antidotes or neutralising remedies against each miasmatic disease.

Hitherto we have been engaged with those cases only of infection of the blood which are induced by miasms, and by animal, vegetable, or mineral poisons; and we have found that, although it may reasonably be supposed that in these intoxications the composition of the circulating mass undergoes certain but yet unknown modifications, it must be confessed that the supposition has not yet been realised, or even sufficiently examined by experimental research. It remains therefore for us to allude to those physical and chemical changes of the blood which have been and may be daily observed, and to ascertain in what diseases these changes take place. When a disease is slowly developed, under the influence of causes, which seriously affect the function of nutrition, and when it repeats, as it were, itself in several organs or tissues, and thus becomes an evil affecting the whole system, it is probable that it has its source in some alteration of one of the two fluids diffused over the whole body; to wit, either of the lymph or of the blood. Let us consider for a moment how much the human constitution is affected by those hygienic circumstances amid which they live; and we can have no difficulty in understanding the reason why an excess or exaggeration of any of these may, in the course of time, bring on certain diseases, and why these diseases must necessarily commence by an alteration of the fluids.

If the privation of the sun's light, or exposure to a cold damp climate, or a deficient or unwholesome food, or want of exercise, and excess of sleep, have the effect of blanching the human body, and of rendering it feeble and apathic, in consequence of the white juices becoming superabundant, and of the red globules of the blood becoming too scanty, it must be evident that the union of all these health-destroying agencies, or an extreme degree of any one of them, must give rise to diseases characterised by a poor thin state of the blood, by the predominance of the lymphatic juices, a deficiency of sensibility, and an imperfection of nutrition; and that these diseases will be slow, chronic, little affected by remedial measures, and curable, rather by the adoption of such hygienic rules as are opposed to those which were the exciting or morbific causes than by any exclusively medical treatment. The chief indication, in short, in such cases, is to "remake" the nutrition of all the tissues of the body. In the list of diseases which are referable to the class now described, we may enumerate the different forms of scrofula, also phthisis pulmonalis, and all tubercular affections. Perhaps, however, we are now advancing too far upon debatable, or at least uncertain ground; for it must be acknowledged that we do not yet know whether the blood or whether the lymph be primarily and chiefly affected in these diseases; and besides, that they will not unfrequently appear even under the most favorable hygienic circumstances. But as the solids are always simultaneously more or less changed in such cases, it may be as well, at least for the present, to exclude these cases from that division of diseases which depend upon, or are necessarily associated with, an altered state of the blood; for as yet we but most imperfectly know whether the changes of this vital fluid are primary and causal, or secondary and consecutive. We have alluded to the effects of those agents which deteriorate, and, as it were, dilute the condition of the blood; let us now, for a moment, consider the oppositely-acting agents. Suppose that a man be exposed to a burning sun, that his appetite be keen and vigorous, that he breathes a pure mountain air, that he sleeps moderately, and takes a due proportion of active corporeal exertion, his blood will in all probability become more rich with red globules, and will be consequently more stimulating, the circulation will become more brisk, the skin will assume a more animated hue, and all the sensibilities, both of mind and body, will be exalted. The general character of the diseases, with which he will be affected, will be those dependant upon plethora, such as violent fevers, inflammations, and congestions; and their course will generally be rapid, requiring for their arrest powerful depletion, and every measure which may diminish the quantity, and impair the energy of the morbid stimulus. But in this, and in all similar cases, an exclusively humoral view of the malady will be incorrect and fallacious; for the existing alterations of the blood are in a manner effaced or overpowered by the predominant influence, which the changes in the solids almost always assume at the same time. We are not however to lose sight of the first set altogether. We have already alluded to the important part it plays in the production of certain pyrexial diseases, especially in hæmoptysis, rheumatism, and gout, in which the fluids are unquestionably often as much at fault as the solids. Still we ought not to attend to one to the exclusion of the other; the influence of both must be duly considered alike in our reasonings upon the etiology and in our treatment of these maladies.

What has been said above on the subject of tuberculous disease, is equally applicable to scorbutus and hæmicelanosis:—they are slowly and very gradually developed; the causes which produce them modify step by step the functions of nutrition; they involve almost every tissue of the body; and they are influenced more by hygienic than by therapeutic measures. But still the nutritive functions are less seriously affected in these diseases than in such as are truly tubercular; and hence the main indication of treatment is rather to modify than to remake them. We are yet quite in the dark as to the real nature of the alterations in the state of the blood, which constitute, or at least invariably accompany them; but to judge by its physical characters, we have reason to believe that it has lost in some degree the force of that affinity which keeps its molecules closely together.

From the preceding train of observation we are led to deduce some interesting conclusions; and one of the most instructive of these is the two-fold classification of all blood-alteration diseases. In the first, we comprise those affections which result from the positive introduction into the system either of an obvious poison, whether that be of an animal, vegetable, or mineral nature; or of the morbific principle of miasms, whether the introduction be effected by injection into the veins, by inoculation, by cutaneous or pulmonary absorption; and in the second are included all the chronic alterations of the blood, in which the chemical composition of this fluid is evidently affected, and which arise chiefly from the influence of certain hygienic conditions, involving and modifying, in a particular degree, the functions of assimilation and of nutrition.

The preceding observations from the pen of M. Roche, published in our valuable cotemporary, deserve not only the perusal, but the study of all our readers. Every calm observer of medical science must have been long satisfied, that the pathological doctrines of the solidists, or mere anatomists, have been of late becoming more and more unstable, and have been forced to abate not a little of that predominant and exclusive importance, which some years ago were almost universally conceded to them. It is, indeed, not very creditable, either to the truth of medical science or to the discretion of its professors, that any particular system of tenets should be unhesitatingly and submissively received, in reference to a machine so curiously and complexly put together as that of the animal body. We venture to predict, that no single or exclusive general doctrine of pathology ever will, or can be altogether and universally true; no matter whether the doctrine refers to the solids or fluids of the living system, or whether it is based upon chemical, mechanical, or electric foundations. A medical philosopher must ever bear in mind, that no one set of phenomena can ever, with propriety, be admitted as the only data or elements for sound pathological reasoning, to the neglect of all the others. It is for this reason that our own mind has always been impressed with the conviction, that the anatomical pathologists of the last 30 years have taken a partial and limited view of the science of disease, and that the edifice which they have reared with so much laudable zeal would lose strength and stability with age, and, although never entirely forgotten, would in course of time be but a portion of a mightier structure, which should be based upon, not one, but a multitude of foundations.—REV.

